# Unlocking phenotypic plasticity provides novel insights for immunity and personalized therapy in lung adenocarcinoma

**DOI:** 10.3389/fgene.2022.941567

**Published:** 2022-09-06

**Authors:** Feng Wang, Hongjuan Du, Bibo Li, Zhibin Luo, Lei Zhu

**Affiliations:** ^1^ Department of Oncology, Chongqing General Hospital, Chongqing, China; ^2^ Department of Thoracic Surgery, Shanghai Pulmonary Hospital, School of Medicine, Tongji University, Shanghai, China

**Keywords:** unlocking phenotype plasticity (UPP), LUAD, drug sensitivities, immune, prognostic

## Abstract

**Background:** Unlocking phenotype plasticity (UPP) has been shown to have an essential role in the mechanism of tumor development and therapeutic response. However, the clinical significance of unlocking phenotypic plasticity in patients with lung adenocarcinoma is unclear. This study aimed to explore the roles of unlocking phenotypic plasticity in immune status, prognosis, and treatment in patients with lung adenocarcinoma (LUAD).

**Methods:** Differentially expressed genes (DEGs) and clinical information of UPP were selected from the cancer genome atlas (TCGA) database, and the GO, KEGG enrichment analyses were performed. The independent prognostic genes were determined by univariate and multivariate Cox regression, and the UPP signature score was constructed. Patients with LUAD were divided into high- and low-risk groups according to the median of score, and the immunocytes and immune function, the gene mutation, and drug sensitivities between the two groups were analyzed. Finally, the results were validated in the GEO database.

**Results:** Thirty-nine significantly DEGs were determined. Enrichment analysis showed that UPP-related genes were related to protein polysaccharides and drug resistance. The prognostic results showed that the survival of patients in the high-risk group was poorer than that in the low-risk group (*p* < 0.001). In the high- and low-risk groups, single nucleotide polymorphism (SNP) and C > T are the most common dissent mutations. The contents of immune cells were significantly different between high- and low-risk groups. And the immune functions were also significantly different, indicating that UPP affects the immunity in LUAD. The results from TCGA were validated in the GEO.

**Conclusion:** Our research has proposed a new and reliable prognosis indicator to predict the overall survival. Evaluation of the UPP could help the clinician to predict therapeutic responses and make individualized treatment plans in patients with LUAD.

## Introduction

Lung cancer is a malignancy with the highest mortality and the second high incidence worldwide (Al-Dherasi et al., 2021; Zheng et al., 2021). Lung cancer mainly includes non-small cell lung cancer (NSCLC) and small cell lung cancer (SCLC). LUAD is one of the main subtypes of lung cancer. However, most patients with LUAD were usually diagnosed at advanced stages. EGFR-TKIs were the primary treatment for patients with EGFR sensitive mutation. However, about 20–30% of patients with EGFR mutant NSCLC have immediate resistance to EGFR-TKIs (Shi et al., 2022). The mechanism of the EGFR-TKI immediate resistance is still not fully clarified. LUAD is a group of mutant types of diseases, and patients in the same pathological stage may have different prognoses, so it is necessary to explore accurate and hopeful biomarkers to help clinicians promote the accuracy and early diagnosis of LUAD and improve the survival of and guide personalized therapy (Yoshizawa et al., 2011; Gandhi et al., 2018).

Phenotype plasticity means that genotypes produce different phenotypes under different environmental conditions and is a crucial mechanism to adapt to environmental heterogeneity. Although researchers have always believed that these biological characteristics have been plaguing this biometric character. However, this point of view has been controversial. Traditionally, phenotype plasticity is considered to be decentralized and differentiated during tissue regeneration or wound healing. Although the degeneration process is the main link of the organization, the decentralization itself has the risk of cancer. Therefore, phenotype plasticity provides a new paradigm to understand the occurrence, development of cancer, and resistance to treatment.

Plasticity exists in various fields of life, and the role of phenotypic plasticity is still seldom studied in mammalian (Matesanz et al., 2021). Recently, in January 2022, the Cancer Discovery released the third edition of Hallmarks of Cancer to explain the mechanism of occurrence, development, and treatment of response characteristics in malignant tumors (Haan, JC et al., 2022). Four new tumor iconic features were introduced based on the previous version, including unlocking phenotypic plasticity, which contributed to a unique point of view. In this study, we aimed to use the clinical, genomic, and transcriptome data of TCGA for prognosis and bioinformatics analysis, to clarify the predictive significance of UPP on the prognosis of LUAD patients and the relationship between UPP and immunity and treatment. This study provided a new insight for the prognosis and treatment of LUAD.

## Materials and methods

### Patient and data acquisition

The LUAD tissue sample was downloaded in the TCGA dataset (https://cancergenome.nih.gov/). TCGA provides 522 clinical, 569 genomes and mutated data, and the Illumina Hi-SEQ RNA SEQ platform provides 594 RNA sequencing (RNA SEQ) data. First, the expression of the unlocked phenotype plasticity-related gene is screened from the gene expression files for differential expression analysis. Then, the differentially expressed genes (DEGs) were analyzed by GO and KEGG. Finally, the data were analyzed for prognosis, immunity, drug resistance, and so on.

### Analysis of DEGs

The key step is to obtain DEGs of the unlocking phenotype plastic-related genes between tumors and normal samples. This study used “limma” package (http://www.bioconductor.org/) to calculate the DEGs (Liu et al., 2021). Wilcox test was performed to obtain DEGs between tumor and normal samples with the standard of | logFC | > 1 and false discovery rate (FDR) value <0.05. Then, the heatmap of DEGs was obtained to visualize their expression in different samples through the “ggplots” package in R. The longitudinal axis is displayed, and the color is used to distinguish between different differential expressions.

### The verification of unlocking phenotypic plasticity-related genes by quantitative real-time polymerase chain reaction

To validate the expression profiles of UPP-related genes in LUAD, we performed quantitative real-time polymerase chain reaction (qRT-PCR) using the A549 cells and normal human bronchial epithelial (NHBE) cells. First, cells were cultured in DMEM (high glucose) + 10% fetal bovine serum +1% penicillin–streptomycin solution. Second, the A549 and NHBE cells were harvested when the confluence of cells is more than 90%. Then, cells were lysed with TRIzol reagent (Invitrogen, CA, United States). The concentrations of the total RNA of A549 and NHBE cells were measured by the NanoDrop 2000 spectrophotometer (Thermo Fisher Scientific, United States), and were synthesized into cDNA using the PrimeScript™ RT reagent kit (Takara, Japan). The qRT-PCR was conducted in the PCR apparatus (Applied Biosystems, Singapore) following the conditions: predenaturation at 95°C for 2 min, 95°C for 15 s, 60°C for 30 s, and 72°C for 30 s. Finally, the gene expression levels were calculated by the 2–Δ Ct method. The primers of genes were designed and synthesized by Sangon Biotech (Shanghai) Co., Ltd., and are available in Supplementary Table S1.

### GO and KEGG enrichment analysis

The DEGs were analyzed by GO analysis through the “ClusterProfiler” package in R software. GO aims to solve the problem of inconsistent gene description in different databases with strictly defined concepts. GO function annotation mainly annotates and classifies differential genes according to the biological process (BP), molecular function (MF), and cellular component (CC). The *p* < 0.05 and FDR <0.05 of the DEGs were used as the localization conditions to obtain the GO item with the highest correlation with the DEGs. The KEGG path with *p*-value <0.05 and at least five genes were selected as the enrichment condition of DEGs. Finally, the bubble map is drawn by the “ggplot”s package in the R software.

### Prognosis-related analysis

We use univariate and multivariate Cox regression analyses to analyze the overall survival (OS) to determine the significant correlation phenotype plasticity-related gene prognosis significantly associated with LUAD. Survival analysis was performed between high- and low-risk groups, and the results were visualized by the Kaplan–Meier curve. To explore the two groups of genetic mutations, we also draw a waterfall map.

### Unlocking phenotypic plasticity and immunity and drug sensitivity prediction

SsGSEA was used to evaluate the difference of immune cell content and function between high- and low-risk groups. *p*-value <0.05 and FDR <0.05 are considered statistically significant. “c2. cp.kegg.v7.1. Symbol” was set as the reference. “McPCounter” is an R-Package that quantifies the absolute abundance of eight immunocytes and two matrix cells using transcription group data. The Genomics of Drug Sensitivity in Cancer database (GDSC; https://www.cancerrxgene.org/) was used to estimate the sensitivity of each patient to chemotherapeutic drugs. The half-maximal inhibitory concentration (IC50) was quantified via the “pRRophetic” package in R (Geeleher et al., 2014).

### Statistical analysis

OS is defined as the time from the diagnosis of LUAD to the patient’s death or last follow-up. “Survival” package was used to draw the Kaplan–Meier survival curve, calculate the hazard ratio (HR), and evaluate the 95% confidence interval (CI) in R. Comparisons between two groups were calculated *via* Wilcoxon rank-sum test. Chi square test or Fisher exact test was used to compare categorical variables. *p* < 0.05 was considered to be statistically significant. All statistical analyses were performed in R (version 4.1.1).

## Results

### DEG screening and heat map

**FIGURE 1:**
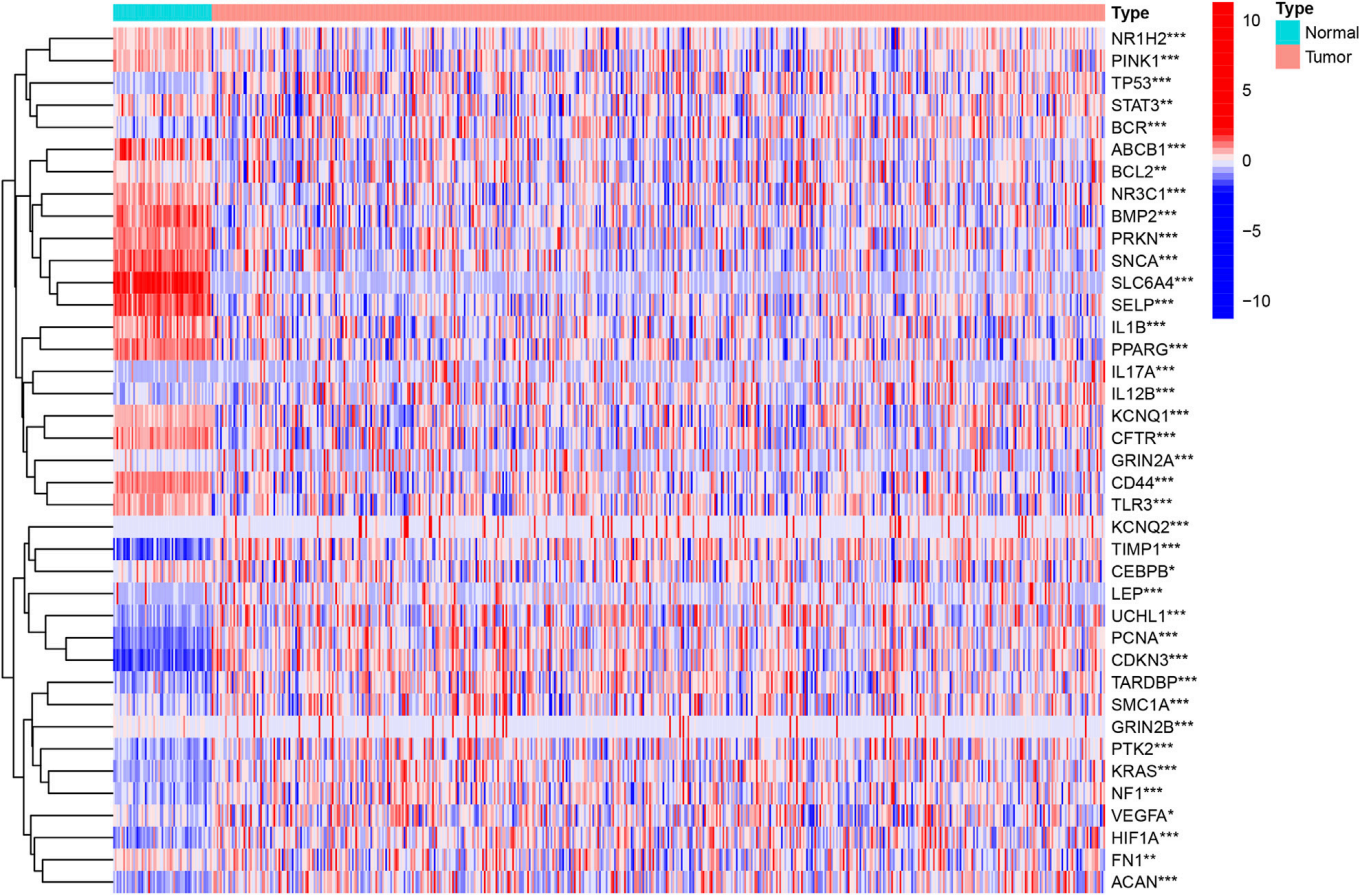
Identification of the UPP-related DEGs. The heatmap analysis of the top 39 DEGs between the tumor and normal samples.

At present, there are a few researches about the unlocking phenotype plasticity genes. In order to clarify the difference of the gene transcription level of unlocking phenotypic plasticity, we obtained DEGs between tumor samples and normal samples using the data of expression profile. A total of 39 significant DEGs were retrieved, of which 20 genes were significantly up-regulated in tumor samples; 19 genes were significantly up-regulated in normal samples. Then, we cluster DEGs and visualize them in the heat map (Figure 1).

**FIGURE 2:**
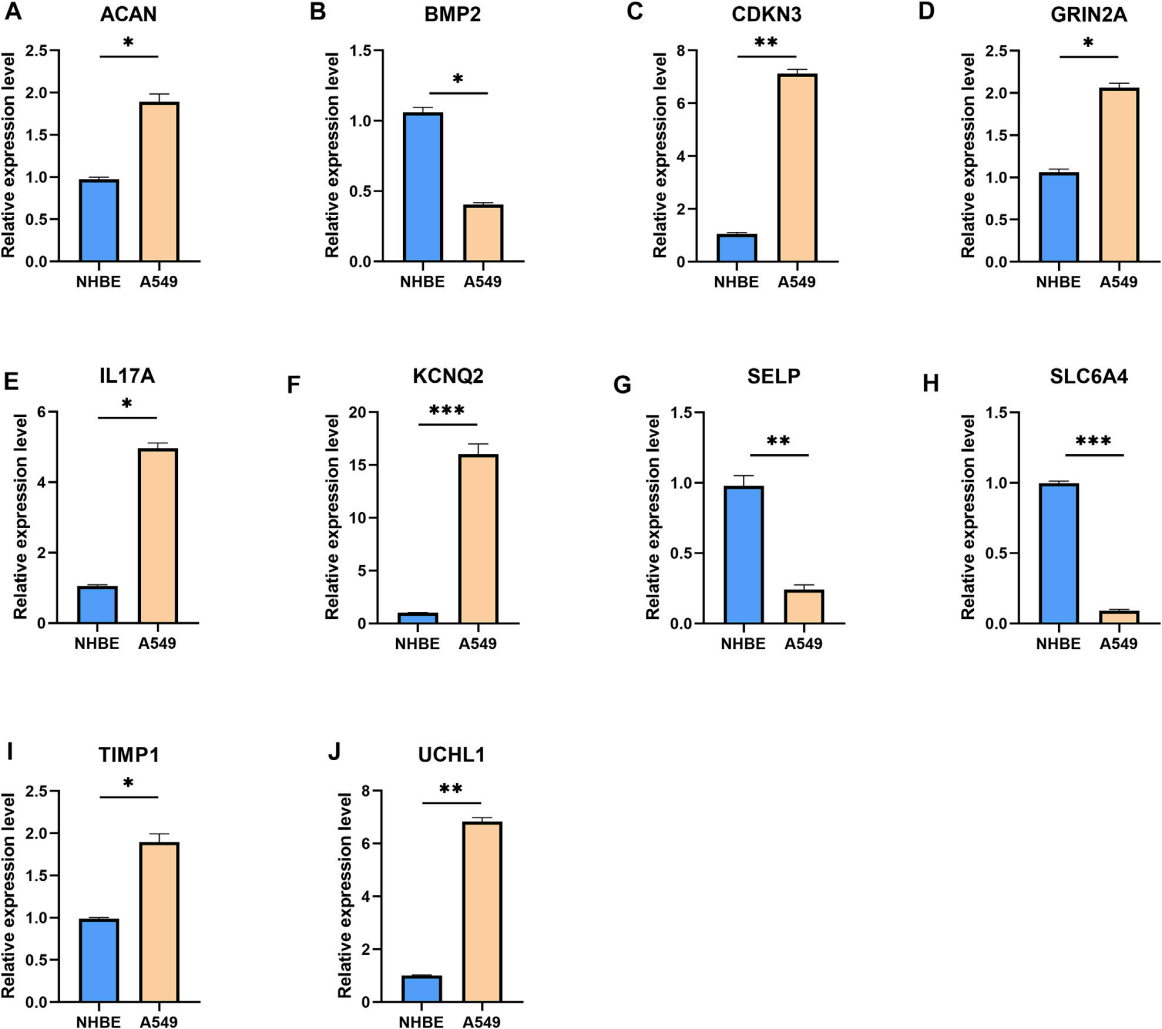
qRT-PCR results for the top 10 genes with the most significant expression differences. The mRNA expression levels of ACAN **(A)**, BMP2 **(B)**, CDKN3 **(C)**, GRIN2A **(D)**, IL17A **(E)**, KCNQ2 **(F)**, SELP **(G)**, SLC6A4 **(H)**, TIMP1 **(I),** and UCHL1 **(J)**. Expression levels of the 10 genes were normalized against GAPDH expression. *p < 0.05, **p < 0.01, ***p < 0.001.

To further validate the UPP expression levels in LUAD, we selected the top 10 genes with the most significant expression differences to perform qRT-PCR, as described above. The results showed that ACAN (Figure 2A), CDKN3 (Figure 2C), GRIN2A (Figure 2D), IL17A (Figure 2E), KCNQ2 (Figure 2F), TIMP1 (Figure 2I), and UCHL1 (Figure 2J) were significantly up-regulated in lung adenocarcinoma cells. However, BMP2 (Figure 2B), SELP (Figure 2G), and SLC6A4 (Figure 2H) were significantly down-regulated in lung adenocarcinoma cells compared with NHBE cells. Collectively, these findings strongly suggested that UPP-related gene expressions were disturbed in LUAD.

### GO and KEGG enrichment analyses

**FIGURE 3:**
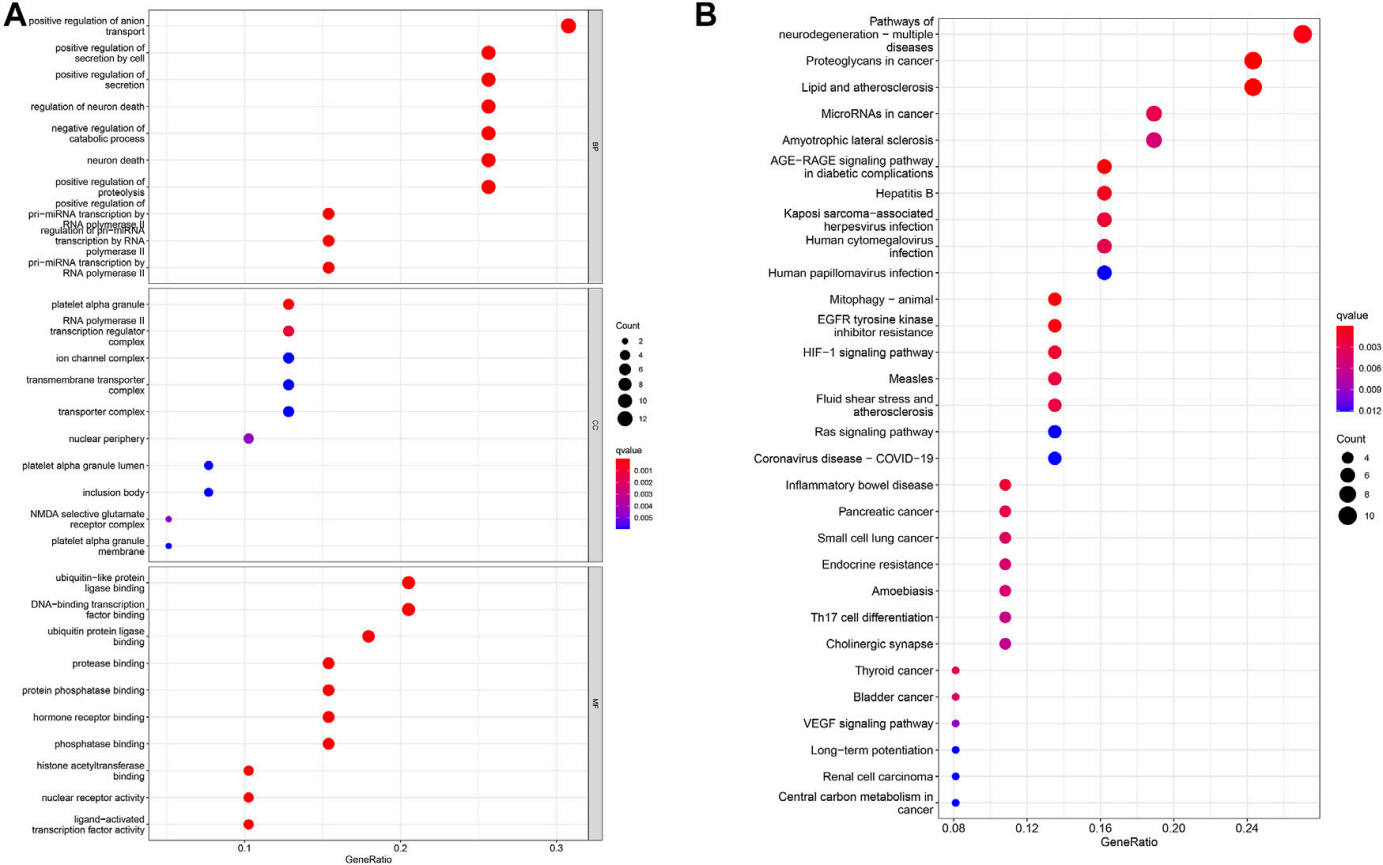
Analysis of DEG distribution and function in LUAD patients. **(A)** GO enrichment analysis of biological process (BP), cellular component (CC) and molecular function (MF) results ranked by the adjusted *p*-value. **(B)** KEGG enrichment analysis results showed that DEGs were strongly associated with the AGE-RAGE signaling pathway and EGFR tyrosine kinase inhibitor resistance pathways.

We use the gene expression of the ClusterProfiler in the R software to perform GO enrichment analysis. The results of BP analysis of GO enrichment suggest that DEGs are mainly enriched in the positive regulation of anion transport, the positive regulation of secretion, and the positive regulation of proteolysis. The CC results showed that DEGs are mainly enriched in the ion channel complex, transmembrane transporter complex, and cation channel complex; MF results showed that DEGs are mainly related to ubiquitin-like protein ligase binding, signaling receptor activator activity, histone acetyltransferase binding, ion transport, cell cycle regulation, and cell adhesion (Figure 3A).

Subsequently, the KEGG pathway enrichment analysis was carried out, and we only showed the first 10 pathways (Figure 3B). These DEGs are strongly correlated with the AGE–RAGE signaling pathway, EGFR tyrosine kinase inhibitor resistance, and other signaling pathways. It has an important impact on cancer progression through important biological processes related to drug resistance.

### Survival analysis

**FIGURE 4:**
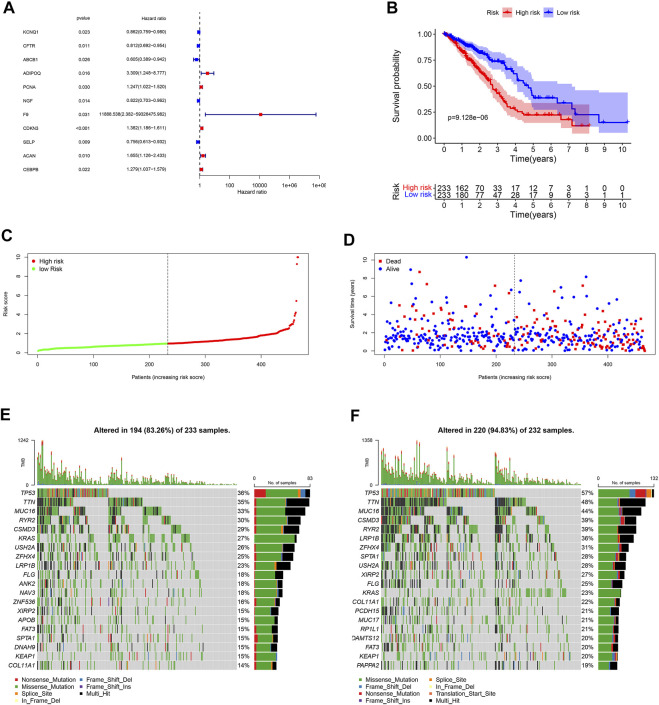
Landscape of UPP and prognosis in LUAD. **(A)** 11 UPP-related genes were obtained by univariate Cox analysis. **(B)** Survival analysis between high- and low-risk groups. **(C)** The risk score curve of all LUAD patients in the TCGA. **(D)** The scatter plot of LUAD survival time periods in the TCGA. **(E)** The Oncoplot of the low-risk group in LUAD. **(F)** The Oncoplot of the high-risk group in LUAD. Oncoplot shows the list of top 20 genes ordered by the number of samples with the gene variants, and the percentage represents the ratio of samples with gene variation to total samples.

Eleven unlocked phenotype plastic-related genes (Figure 4A) were screened by univariate Cox analysis. Next, multivariate Cox analysis results showed that ABCB1, ADIPOQ, NGF, F9, CDKN3, ACAN, and CEBPB are independent prognostic genes. According to the expression level and coefficient of the separate prognostic gene, we constructed the signature following the formula: Risk Score = (1.267 × ADIPOQ) - (0.523 × ABCB1) - (0.233 × NGF) + (12.988 × F9) + (0.281 × CDKN3) + (0.605 × ACAN) + (0.168 × CEBPB). The samples were divided into two groups according to the median of the risk score (Table 1). Survival analysis was conducted for high- and low-risk groups. Compared to the low-risk group, patients in the high-risk group had worse prognoses (*p* < 0.01) (Figures 4B–D).

**TABLE 1:** Independent prognostic genes and coefficients.

Gene	Coef
ABCB1	−0.523
ADIPOQ	1.267
NGF	−0.233
F9	12.988
CDKN3	0.281
ACAN	0.605
CEBPB	0.168

### Mutations in high and low groups

**FIGURE 5:**
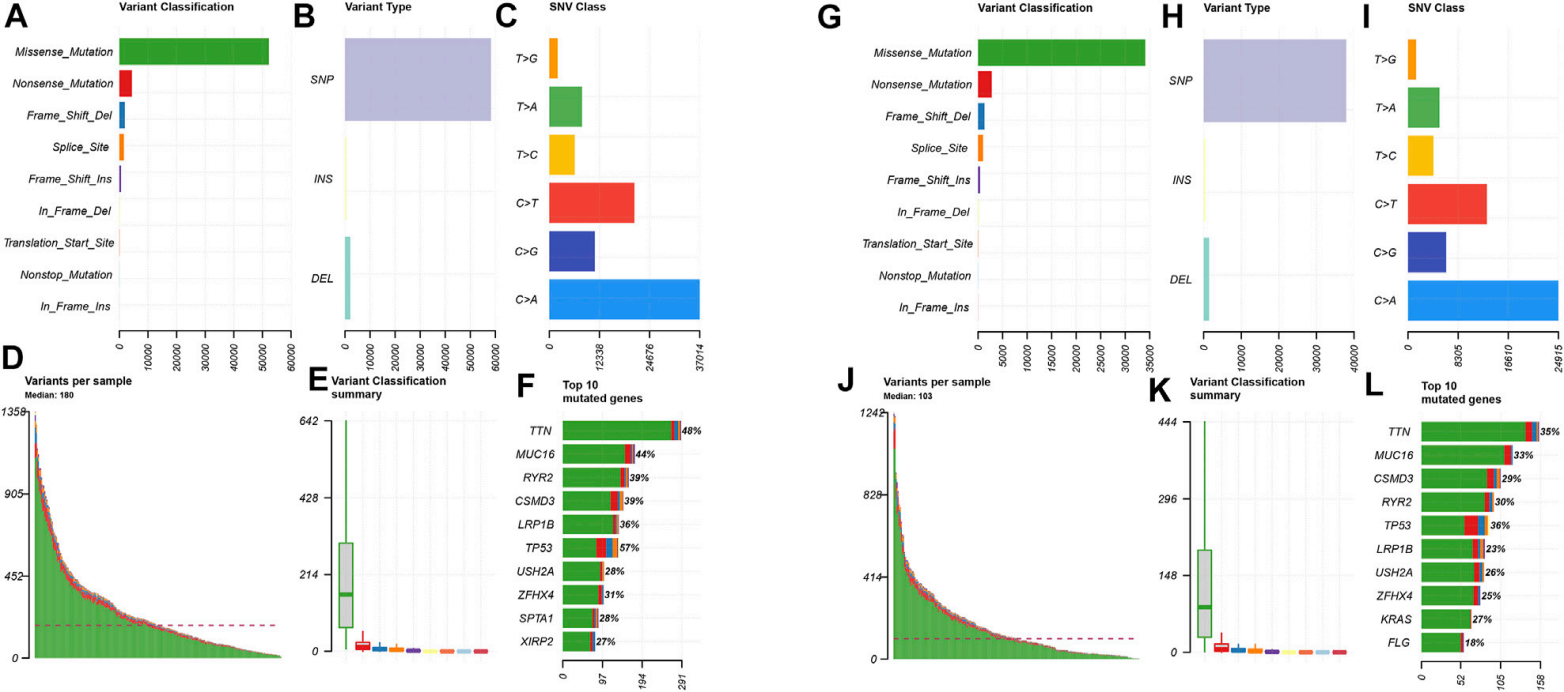
Distributions of mutant genes in high- and low-risk groups. **(A)** Variant classification and frequency of gene mutations in the high-risk group. **(B)** Variant type in the high-risk group. **(C)** Frequency of SNV classes in the high-risk group. **(D)** Median of variants per sample in the high-risk group. **(E)** Variant classification summary in the high-risk group. **(F)** List of top 10 mutated genes in the high-risk group. **(G)** Variant classification and frequency of gene mutations in the low-risk group. **(H)** Variant type in the low-risk group. **(I)** Frequency of SNV classes in the low-risk group. **(J)** Median of variants per sample in the low-risk group. **(K)** Variant classification summary in the low-risk group. **(L)** List of top 10 mutated genes in the low-risk group.

In order to explore the cases of the genetic mutation of the two groups, the waterfall map was drawn. High- and low-risk groups are shown in Figures 3E,F, respectively (Figures 4E,F). TP53, TTN, and MUC16 also have a higher mutation rate. The top 20 mutation genes, PCDH15, MUC17, RPIL1, DAMTS12, and PAPPA219 mutations, exist only in the high-risk group, while ANK2, NAV3, ZNF536, APOB, and DANH9 mutations exist only in the low-risk group. Single nucleotide polymorphism (SNP) was responsible for such variants, and single nucleotide variants (SNVs) mostly occurred as C > A and C > T in the high- (Figures 5A–F) and low-risk (Figures 5G–L) groups.

### Unlocking phenotypic plasticity and immune correlation

**FIGURE 6:**
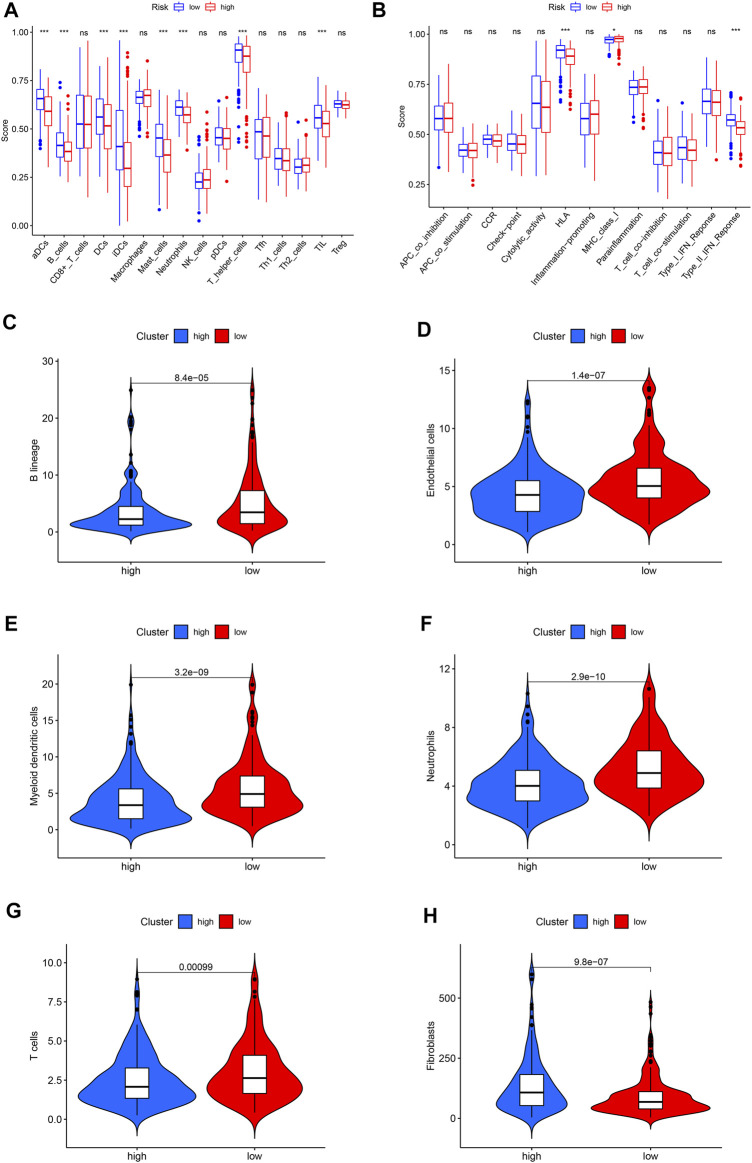
The landscape of immune infiltration in LUAD. **(A)** Differences of immune cells in the high- and low-risk groups. **(B)** Differences in the immune function between high- and low-risk groups. **(C)** Violin plot of B lineage. **(D)** Violin plot of endothelial cells. **(E)** Violin plot of myeloid dendritic cells. **(F)** Violin plot of neutrophils. **(G)** Violin plot of T cells. **(H)** Violin plot of fibroblasts. The horizontal line in the Violin plot represents the median, and blue and red represent the high-risk and low-risk groups, respectively. *p* < 0.05 shows the significant statistical difference between two groups.

Immunocytes in tumor environments play important roles in tumor progression. We use ssGSEA to assess the correlation between immunocytocytes and related functions. The immune cells aDCs, B_cells, DCs, iDCs, Mast_cells, Neutrophils, T_helper_cells, and TIL have significant differences between two groups. The immune function is significantly different in HLA, MHC_class_I, and Type_II_IFN_Reponse (Figures 6A,B). The absolute abundance of eight immune cells and two stromal cells was evaluated using MCPcounter. The results showed that the abundance of B lineage, endothelial cells, myeloid dendritic cells, neutrophils, and T cells was higher in the low-risk group (Figures 6C–G), while the abundance of fibroblasts was higher in the high-risk group (Figure 6H).

### Relations between unlocking phenotypic plasticity and therapeutic sensitivity

**FIGURE 7:**
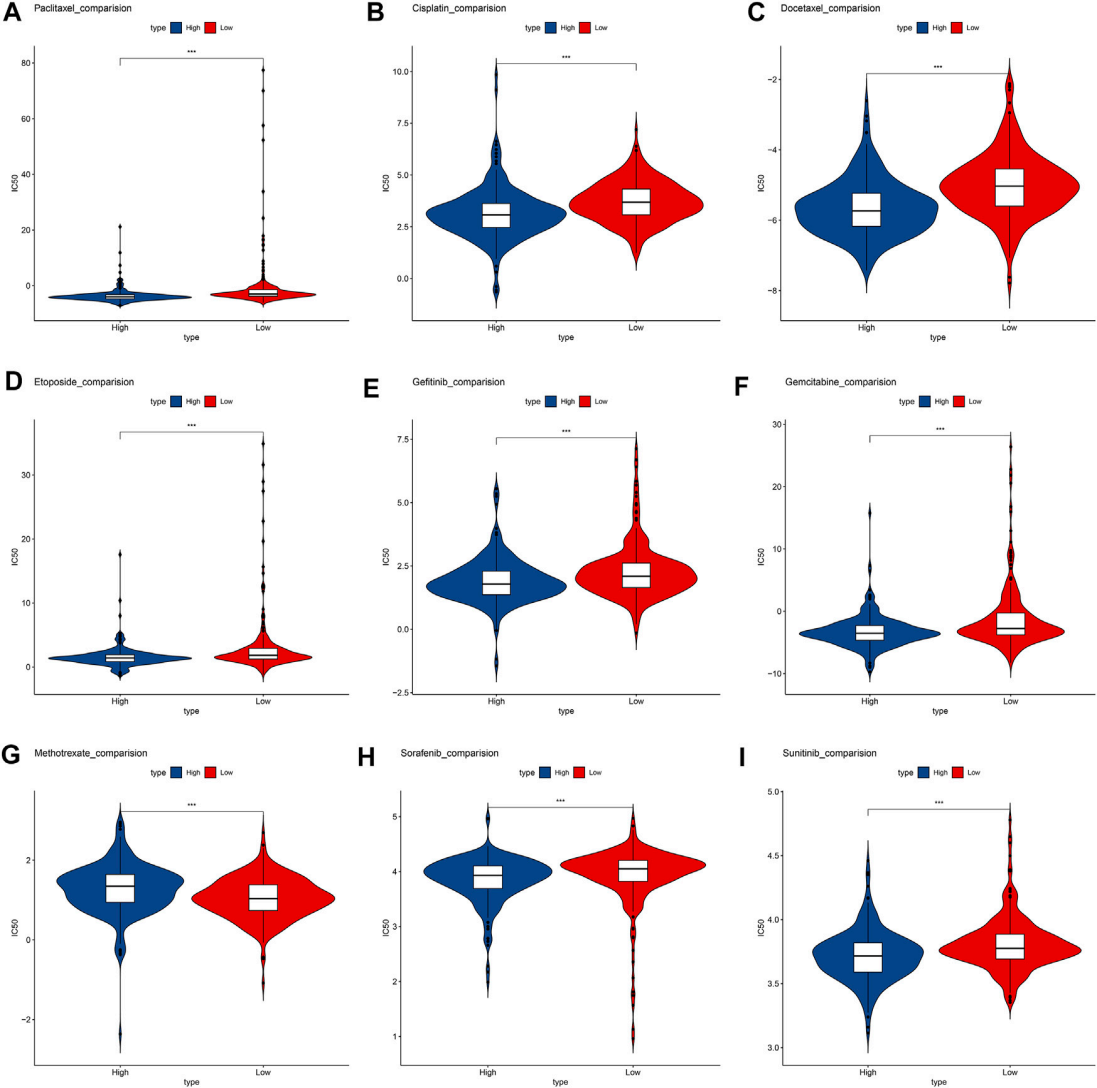
Box plots depicted the differences in the estimated IC50 levels of **(A)** Paclitaxel; **(B)** Cisplatin; **(C)** Docetaxel; **(D)** Etoposide; **(E)** Gefitinib; **(F)** Gemcitabine; **(G)** Methotrexate; **(H)** Sorafenib; **(I)** and Sunitinib between the high- and low-risk groups.

We compare the commonly used chemotherapy drugs, including paclitaxel (Figure 7A), cisplatin (Figure 7B), docetaxel (Figure 7C), etoposide (Figure 7D), gefitinib (Figure 7E), gemcitabine (Figure 7F), methotrexate (Figure 7G), sorafenib (Figure 7H), and sunitinib (Figure 7I)-estimated IC50 levels. Our data showed that the IC50 level of methotrexate in the low-risk group is significantly lower than that in the high-risk group, indicating that patients in the low-risk group are more sensitive to methotrexate. On the contrary, paclitaxel, cisplatin, docetaxel, etoposide, gefitinib, gemcitabine, sorafenib, and sunitinib were more sensitive in the high-risk group.

### External verification

**FIGURE 8:**
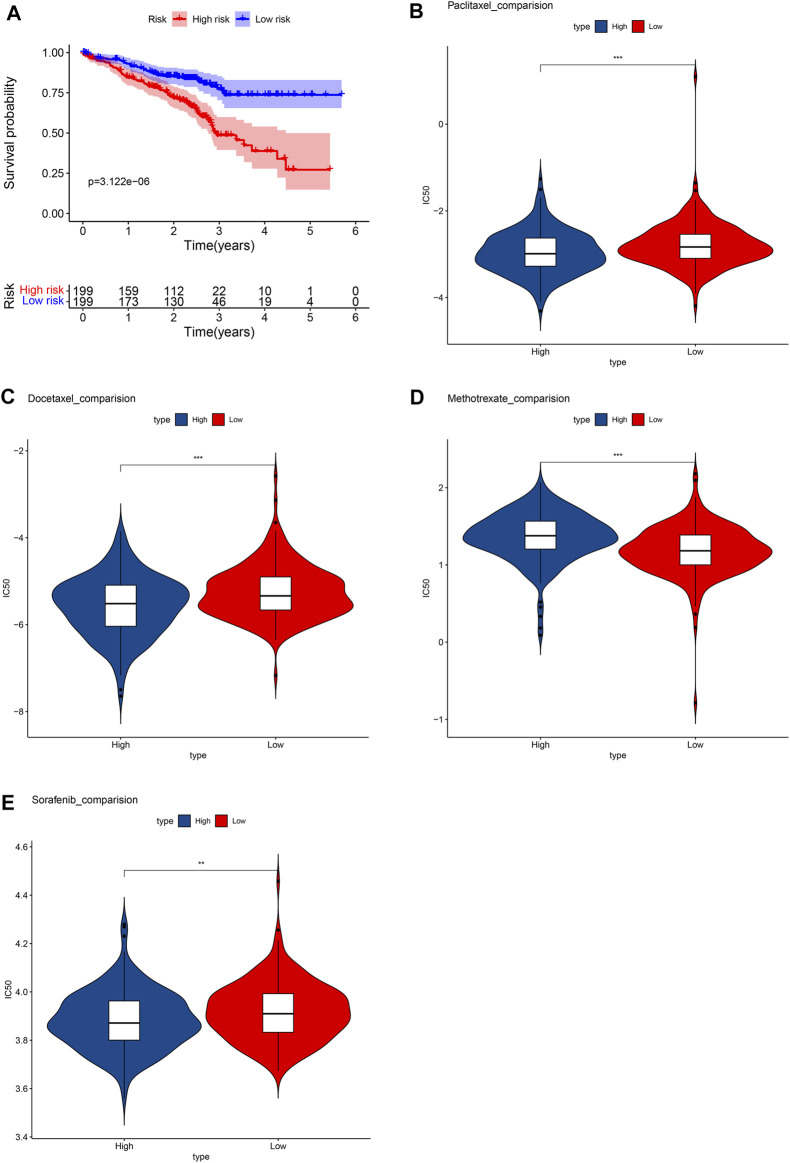
Verify the results in the GEO database. **(A)** Survival analysis between high- and low-risk groups. Box plots depicted the differences in the estimated IC50 levels of **(B)** Paclitaxel; **(C)** Docetaxel; **(D)** Methotrexate; **(E)** and Sorafenib between the high- and low-risk groups.

In the GSM72094 dataset of GEO database, we further verified the effectiveness of unlocking phenotypic plasticity score in predicting prognosis and drug sensitivity. Consistent with the results of the TCGA database, the prognosis of the high-risk group was significantly worse than that of the low-risk group in GSM72094 dataset (Figure 8A). In addition, the drug sensitivity of the prognostic score was further evaluated to speculate on the therapeutic benefits of LUAD patients. The results showed that the low-risk group was more sensitive to methotrexate, while the high-risk group was more sensitive to paclitaxel, docetaxel, and sorafenib (Figures 8B–E), which was consistent with the drug sensitivity results in the TCGA database.

## Discussion

Previous findings have shown that phenotypic plasticity is directly related to the origin, progression, and treatment response of cancer cells (Healy and Schulte, 2015). The environmental factors affecting phenotype can be continuous or discontinuous, and the influence of environment can last for the whole life cycle of organism. Tumor heterogeneity stems, in part, from the ability of cancer cells to switch between phenotypic states, but the genetic of this cellular plasticity is still poorly understood.

In this study, we excavated the public database TCGA to explore the influence of unlocking phenotypic plasticity on the survival of LUAD patients, which proves that there is a worse clinical outcome in patients with unlocked phenotype plastic-related genetic mutations. The effect mechanism of unlocking the phenotypic plasticity on LUAD was discussed by bioinformatics for the first time. In what ways does unlocking the phenotypic plasticity affects the prognosis of LUAD patients? In this study, we were analyzed by the differential expression of the unlocked population plasticity and obtained 39 DEGs. GO and KEGG analyses were performed on DEGs to find the possible functions of DEGs and the metabolic and signaling pathways mainly involved.

The results of GO showed that the DEGs were related to the positive regulation of anion transport, positive regulation of secretion, positive regulation of proteolysis, and histone acetyltransferase binding. Studies have found that the most distinct group of protein modifiers is histone acetyltransferase (HATS). Most groups of histone acetylationase have substrate specificity to guide the acetylation of a particular residue within one or more core groups (Salutari et al., 2022). However, these substrate specificities are not fixed and can be changed by catalytic subunits and protein complexes. The presence of many of HAT complexes expands the modified state of the chromosome template. Therefore, more and more evidence suggests that specific cellular processes are related to the precise model of histone modification (Zhao et al., 2022). Ion transport, protein catabolism, and other functions are also indispensable physiological mechanisms for tumor cell proliferation and metastasis. Tumor microenvironment is very important for the heterogeneity and the plasticity of tumor cells of LUAD.

We found that DEGs were mainly enriched in AGE-RAGE signaling pathway, and EGFR tyrosine kinase inhibitor resistance. The AGE-RAGE signal pathways promote autophagy flux while inhibiting apoptotic signals in cancer cells (Waghela, BN et al., 2021). The activation of autogenesis, such as beclin-1 passes through autophagy to promote cancer cell survival (Chhipa et al., 2019). The activation of the AGE-RAGE signal also produces oxygen-free radicals, leading to oxidative stress and activation of NF-κB. The latter secretes proinflammatory cytokines, growth factors, and adhesion molecules, such as intercellular adhesion molecule-1 and vascular cell adhesion molecule-1, which eventually lead to cancer progression. AGEs may change the extracellular matrix (ECM) through the production of cell surface receptors and proinflammatory cytokines. The overexpression of RAGE increases the migration, invasion, and epithelial mesenchymal transformation of human lung adenocarcinoma cells through the ERK signaling pathway (El-Far et al., 2020). Recent reports show that AGEs also promote cell proliferation and migration of breast cancer cells (Chen et al., 2020). Bhargav N. Waghela et al.'s recent studies have shown that AGE-RAGE signaling pathways are related to programmed cell death signal, apoptosis, and autophagy (Waghela, BN et al., 2021).

Although TKI-induced or selected genetic changes can drive drug resistance, drug resistance occurs in tumor cells without genetic changes. In the case of no gene changes, tumor cells are plasticity; from tumors, the various components of the microenvironment causing a change in tumor phenotypes may be the driving factor of drug resistance (Tsai and Nusinov et al., 2019; Gkountakos et al., 2022). A tumor microenvironment (TME) is a mixture of active ingredients and dynamic components, including a large number of metabolites, which interact to promote carcinogenic survival and proliferation. In particular, EGFR-TKI-resistant cell-secreted lactic acid is swallowed by cancer-related fibroblasts (CAFs), triggering the excess secretion of hepatocyte growth factor (HGF) and subsequent MET signal activation, indicating that there is a non-cellular autonomous metabolic EGFR-TkI drug resistance mechanism (Zhao et al., 2019). Recent studies have shown that extracellular matrix (ECM) has played a new role in malignant cancer progression and targeted therapeutic resistance (Levy et al., 2016; Chen W. et al., 2022). Yanan Yang et al. studied ECM as a unique role in obtaining EGFR TKIS drug resistance (Wang, et al., 2018).

Phenotype plasticity in the tumor process is also driven by the activation of the developmental differentiation procedure—epithelial–mesenchymal transition (EMT); an EMT is the broadest example of phenotype plasticity. Its role in tumor progression and metastasis has been fully confirmed. A transfer is the cause of most cancer patients (Yang et al., 2018). Recently, researchers have discovered several transition conditions that occurred during skin squamous cell carcinoma and breast tumors (Rubin, MA et al., 2020). Tumor cells in different differentiation stages, from epithelial to complete mesenchymal cells, exhibited similar tumor proliferation capabilities through intermediate hybridization. Tumor cell subsets show other cell plasticity and invasiveness.

We divide patients into high- and low-risk groups through univariate and multivariate Cox regression analyses, and the results show that the high-risk groups are poorer than the low-risk groups. After the differential expression gene is submitted to the associated prognostic gene, seven intersection genes are obtained, namely, ABCB1, ADIPOQ, NGF, F9, CDKN3, ACAN, and CEBPB. ABCB1 is a member of the ABCB subfamily located on chromosome 7q21. It consists of 28 exons, encoding 1280 amino acid glycoproteins (MDR1/PGP). MDR1/PGP produces different interactions with different drugs (Manna et al., 2015). In addition to a wide range of substrate specificity, the unique feature of MDR1/PGP is its base ATPase activity. MDR1/PGP can output most neutral and cationic hydrophobic compounds, and cancer cells can efficiently utilize this mechanism as the main barrier to chemotherapy. Cells with higher MDR1/PGP levels have selective advantages in adapting to harsh environments such as hypoxia or inflammation. The study found that MDR1/PGP confers cancer cell resistance by inhibiting caspase-dependent apoptosis (Yang et al., 2022). The effectiveness of these interesting conjectures is to be further confirmed and confirmed by experiments such as the knockout model.

Human CDKN3 gene encodes cyclin-dependent kinase inhibitor 3, which is a bispecific protein tyrosine phosphatase of the CDC14 group. CDKN3 is used as CDK1 and CDK2 inhibitory proteins, which are conventionally considered a negative regulatory factor of the cell cycle process (Li et al., 2022). Although CDKN3 has a negative adjustment effect on CDK1 and CDK2, the carcinogenic effect of CDKN3 is abnormally expressed, which is related to a variety of human cancers. In esophageal cancer, CDKN3 affects the progress of cancer by promoting cell cycle and chemotherapy. Chao Fan found that CDKN3 has increased in NSCLC, and the CDKN3 high expression is always related to the total survival of these patients (Fan et al., 2015). There is also evidence to support that CDKN3 in cervical cancer (CC) can not only be used as a useful marker that survives and selects additional chemotherapy or specific targeted cancer treatment but also as a specific small drug for developing anti-CC potential target.

CCAAT/enhancer binding protein (CEBPS) is a leucine zipper transcription factor family to participate in cell proliferation and differentiation (Huang et al., 2020). Although it is well known that CEBPB is a transcription factor involved in adipocytes and immunocyte differentiation. Still, the function of CEBPB in NSCLC has been controversial, which may be because CEBPB depends on the synergistic transcription factor and/or the apparent genetic state of the respective gene sites in the intracellular environment. Studies have shown that under the transcription of CEBPB, long-coded RNA LOC102724169 can enhance cisplatin on the therapeutic effect of ovarian cancer cells (Lynch and May. 2011). CEBPS enhances the drug resistance of cisplatin to cisplatin by enhancing nasopharyngeal carcinoma cells in combination with the serine protease inhibitor Kazal 5-type promoter region. There is also evidence to support CEBPB-NRF2 synergies to drive cancer malignancy by improving the initial tumor activity and drug resistance (Perino et al., 2014).

Introduction to the mutation of high- and low-risk groups, in the top 20 mutation genes, PCDH15, MUC17, RPIL1, DAMTS12, and PAPPA219, exists only in the high-risk group, while ANK2, NAV3, ZNF536, APOB, and DANH9 exist only in the low-risk group. In the high- and low-risk groups, the most common mutation is missense mutation, followed by nonsense mutation. Single nucleotide polymorphism (SNP) was responsible for such variants, and single nucleotide variants (SNVs) mostly occurred as C > A and C > T. However, the role of PCDH15, MUC17, RPIL1, DAMTS12, and PAPPA219 expressed in a tumor microenvironment remains to be studied.

The anti-PD-1 and anti-PD-L1 antibodies have proven effective for certain LUAD patients (Zhang et al., 2020). Their therapeutic response is related to immune infiltration and related gene expression in the tumor environment. Therefore, it is very important to identify immune-related cells in the tumor environment. We found that aDCs, B_cells, DCs, iDCs, Mast_cells, Neutrophils, T_helper_cells, and TIL infiltration levels are related to the low-risk group. The immune function is different in HLA, MHC_class_I, and Type_II_IFN_Reponse. MCPcounter results show that B lineage, endothelial cells, myeloid dendritic cells, neutrophils, and T cells have higher abundance in the low-risk group, while fibroblasts are high in the high-risk groups. These results indicate that patients with the low-risk group may benefit from immune checkpoint inhibitors. Phenotype plasticity may be related to the adjustment of tumor microenvironment into fibroblast abundance, thereby affecting tumor growth and progression.

Although there are more and more treatment programs, in modern cancer medicine, the development of drug resistance is a major challenge and the cause of failure and disease recurrence. LUAD usually has chemotherapy to resist drug resistance (Dokla, EME et al., 2019). Our data show that low-risk patients are more sensitive to methotrexate. Methotrexate combined immunosuppressive treatment may alleviate the drug resistance mechanism. High-risk groups are more sensitive to paclitaxel, cisplatin, docetaxel, etoposide, gefitinib, gemcitabine, sorafenib, and sunitinib. Lowering the abundance of fibroblasts in a tumor microenvironment may be a targeted treatment direction (Chen J. et al., 2022).

Cancer cells have obtained two important malignant characteristics of metastasis and drug resistance during differentiation. The differentiation state of the tumor is a key determinant of therapeutic resistance. It was studied in an experiment that induced EMT or degeneration in a cancer cell line and mouse model (ScheelWeinberg. 2011). The results show that the deplified promotes drug resistance to various chemotherapeutic drugs, and the decimalization increases about 10 times the IC50 dose of chemotherapeutic drugs. This requires further *in vitro* and *in vivo* studies. In addition, further clinical research is needed to determine if phenotype plasticity is of independent prognostic biomarker, as well as their relationship with the therapeutic effect.

We first link the unlocked phenotype plasticity with LUAD. Our study shows that ABCB1, ADIPOQ, NGF, F9, CDKN3, ACAN, and CEBPB may be potential genes for resistance to drug resistance. It may be a useful biomarker that affects the plasticity of the phenotype. Phenotype plasticity may provide potential biomarkers between tumor microenvironments, ICIS, and treatment reactions, which may be valued for LUAD treatment and prognosis.

Similar to previous studies (Yi et al., 2021), we successfully divided patients with lung adenocarcinoma into high-risk and low-risk groups by constructing the UPP-related model through gene signatures. This model can predict the prognosis and evaluate the content of immune cells. In addition, the model also evaluated the function of immune cells, the abundance of stromal cells, and drug sensitivity. Immune cells and this model can predict the sensitivity of patients to chemotherapy drugs and help clinical patients formulate personalized treatment plans.

However, our research still has some limitations: first, the lack of experiments to verify the association between ingredients such as immune cells and prognosis in microtumor environments. Second, the lack of large clinical samples to forward the predictive value of prognosis characteristics of LUAD patients. In addition, the experimental exploration of potential functions and mechanisms in the signal and immunization infiltration of unlocking phenotype plasticity-related genes in LUAD progression. Therefore, further study is required in a clinical trial of larger sample quantities to verify the value of unlocking phenotype plasticity in LUAD prognosis.

## Data Availability

The original contributions presented in the study are included in the article/Supplementary Material; further inquiries can be directed to the corresponding author.

## References

[B1] Al-Dherasi A. Huang Q. T. Liao Y. Al-Mosaib S. Hua R. Wang Y. (2021). A seven-gene prognostic signature predicts overall survival of patients with lung adenocarcinoma (LUAD). Cancer Cell. Int. V21N1, 294. 10.1186/s12935-021-01975-z PMC8183047 34092242

[B2] Chen J. Fu Y. Hu J. He J. (2022b). Hypoxia-related gene signature for predicting LUAD patients' prognosis and immune microenvironment. Cytokine 152, 155820. 10.1016/j.cyto.2022.155820 35176657

[B3] Chen M-C. Chen K-C. Chang G-C. Lin H. Wu C-C. Kao W-H. (2020). RAGE acts as an oncogenic role and promotes the metastasis of human lung cancer. Cell. Death Dis. 11, 265. 10.1038/s41419-020-2432-1 32327633 PMC7181650

[B4] Chen W. Li W. Liu Z. Ma G. Deng Y. Zhu L. (2022a). Identification of tumor microenvironment-based genes associated with acquired resistance to EGFR Tyrosine Kinase Inhibitor in Lung Adenocarcinoma. J. Cancer 13 (3), 877–889. 10.7150/jca.57008 35154456 PMC8824894

[B5] Chhipa A. S. Borse S. P. Baksi R. Lalotra S. Nivsarkar M. (2019). Targeting receptors of advanced glycation end products (RAGE): Preventing diabetes induced cancer and diabetic complications. Pathol. Res. Pract. 215, 152643. 10.1016/j.prp.2019.152643 31564569

[B6] Dokla E. M. E. Fang C. S. Abouzid K. A. M. Chen C. S. (2019). 1, 2, 4-Oxadiazole derivatives targeting EGFR and c-Met degradation in TKI resistant NSCLC. Eur. J. Med. Chem. 2019V182N, 111607. 10.1016/j.ejmech.2019.111607 31446247

[B7] El-Far A. H. Sroga G. Jaouni S. K. A. Mousa S. A. (2020). Role and mechanisms of RAGE-ligand complexes and RAGE-inhibitors in cancer progression. Int. J. Mol. Sci. 21, E3613. 10.3390/ijms21103613 32443845 PMC7279268

[B8] Fan C. Chen L. Huang Q. Shen T. Welsh E. A. Teer J. K. (2015). Overexpression of major CDKN3 transcripts is associated with poor survival in lung adenocarcinoma. Br. J. Cancer 113 (12), 1735–1743. 10.1038/bjc.2015.378 26554648 PMC4701993

[B9] Gandhi L. Rodríguez-Abreu D. Gadgeel S. Esteban E. FelipE De Angelis F. Domine M. (2018). Pembrolizumab plus chemotherapy in metastatic non–small-cell lung cancer. N. Engl. J. Med. 378 (22), 2078–2092. 10.1056/NEJMoa1801005 29658856

[B10] Geeleher P. Cox N. Huang R. S. (2014). pRRophetic: an R package for prediction of clinical chemotherapeutic response from tumor gene expression levels. PLoS One 583 9, e107468. 10.1371/journal.pone.0107468 PMC4167990 25229481

[B11] Gkountakos A. Centonze G. Vita E. Belluomini L. Milella M. Bria E. (2022). Identification of targetable liabilities in the dynamic metabolic profile of EGFR-mutant lung adenocarcinoma: Thinking beyond genomics for overcoming EGFR TKI resistance. Biomedicines 10, 277. 10.3390/biomedicines10020277 35203491 PMC8869286

[B12] Haan J. C. Bhaskaran R. Ellappalayam A. Bijl Y. Griffioen C. J. Lujinovic E. (2022). MammaPrint and BluePrint comprehensively capture the cancer hallmarks in early-stage breast cancer patients. Genes. Chromosom. Cancer 61 (3), 148–160. 10.1002/gcc.23014 34841595 PMC9299843

[B13] Healy T. M. Schulte P. M. (2015). Phenotypic plasticity and divergence in gene expression. Mol. Ecol. 24 (13), 3220–3222. 10.1111/mec.13246 26096949

[B14] Huang Y. Lin L. Shen Z. Li Y. Cao H. Peng L. (2020). CEBPG promotes esophageal squamous cell carcinoma progression by enhancing PI3K-AKT signaling. Am. J. Cancer Res. 10 (10), 3328–3344. PMID:33163273. 33163273 PMC7642652

[B15] Levy B. P. Rao P. Becker D. J. Becker K. (2016). Attacking a moving target: Understanding resistance and managing progression in EGFR-positive lung cancer patients treated with tyrosine kinase inhibitors. Oncol. Willist. Park) 30 (7), 601–612. PMID: 27432364. 27432364

[B16] Li M. Che N. Jin Y. Li J. Yang W. (2022). CDKN3 overcomes bladder cancer cisplatin resistance via LDHA-dependent glycolysis reprogramming. Onco. Targets. Ther. 15, 299–311. 10.2147/OTT.S358008 35388272 PMC8977226

[B17] Liu S. Wang Z. Zhu R. Wang F. Cheng Y. Liu Y. (2021). Three differential expression analysis methods for RNA sequencing: Limma, EdgeR, DESeq2. J. Vis. Exp. VN175. 10.3791/62528 34605806

[B18] Lynch V. J. May G. Wagner G. P. (2011). Regulatory evolution through divergence of a phosphoswitch in the transcription factor CEBPB. Nature 480, 383–386. 10.1038/nature10595 22080951

[B19] Manna I. Gambardella A. Labate A. Mumoli L. Ferlazzo E. Pucci F. (2015). Polymorphism of the multidrug resistance 1 gene MDR1/ABCB1 C3435T and response to antiepileptic drug treatment in temporal lobe epilepsy. Seizure 24, 124–126. 10.1016/j.seizure.2014.09.010 25458099

[B20] Matesanz S. Blanco-Sánchez M. Ramos-Muñoz M. de la Cruz M. Benavides R. Escudero A. (2021). Phenotypic integration does not constrain phenotypic plasticity: Differential plasticity of traits is associated to their integration across environments. New Phytol. 231 (6), 2359–2370. 10.1111/nph.17536 34097309

[B21] Perino A. Pols T. W. Nomura M. Stein S. Pellicciari R. Schoonjans K. (2014). TGR5 reduces macrophage migration through mTOR-induced C/EBPβ differential translation. J. Clin. Investig. 124 (12), 5424–5436. 10.1172/JCI76289 25365223 PMC4348975

[B22] Rubin M. A. Bristow R. G. Thienger P. D. Dive C. Imielinski M. (2020). Impact of lineage plasticity to and from a neuroendocrine phenotype on progression and response in prostate and lung cancers. Mol. Cell. 80 (4), 562–577. 10.1016/j.molcel.2020.10.033 33217316 PMC8399907

[B23] Salutari I. Caflisch A. (2022). Dynamics of the histone acetyltransferase lysine-rich loop in the catalytic core of the CREB-binding protein. J. Chem. Inf. Model. 62 (4), 1014–1024. 10.1021/acs.jcim.1c01423 35119862

[B24] Scheel C. Weinberg R. A. (2011). Phenotypic plasticity and epithelial-mesenchymal transitions in cancer and normal stem cells? Int. J. Cancer 129 (10), 2310–2314. 10.1002/ijc.26311 21792896 PMC3357895

[B25] Shi Y. Xu Y. Xu Z. Wang H. Zhang J. Wu Y. (2022). TKI resistant-based prognostic immune related gene signature in LUAD, in which FSCN1 contributes to tumor progression. Cancer Lett. 532, 215583. 10.1016/j.canlet.2022.215583 35149175

[B26] Tsai C. J. Nussinov R. (2019). Emerging allosteric mechanism of EGFR activation in physiological and pathological contexts. Biophys. J. 117, 5–13. 10.1016/j.bpj.2019.05.021 31202480 PMC6626828

[B27] Waghela B. N. Vaidya F. U. Ranjan K. Chhipa A. S. Tiwari B. S. Pathak C. (2021). AGE-RAGE synergy influences programmed cell death signaling to promote cancer. Mol. Cell. Biochem. 476 (2), 585–598. 10.1007/s11010-020-03928-y 33025314

[B28] Wang Y. Zhang T. Guo L. Ren T. Yang Y. (2018). Stromal extracellular matrix is a microenvironmental cue promoting resistance to EGFR tyrosine kinase inhibitors in lung cancer cells. Int. J. Biochem. Cell. Biol. 106, 96–106. 10.1016/j.biocel.2018.11.001 30471423

[B29] Yang X. Liang X. Zheng M. Tang Y. (2018). Cellular phenotype plasticity in cancer dormancy and metastasis. Front. Oncol. 8, 505. 10.3389/fonc.2018.00505 30456206 PMC6230580

[B30] Yang Y. Teng Q. X. Wu Z. X. Wang J. Q. Lei Z. N. Lusvarghi S. (2022). PBK/TOPK inhibitor OTS964 resistance is mediated by ABCB1-dependent transport function in cancer: *In vitro* and *in vivo* study. Mol. Cancer 21 (1), 40. 10.1186/s12943-022-01512-0 35135547 PMC8822834

[B31] Yi M. Li A. Zhou L. Chu Q. Luo S. Wu K. (2021). Immune signature-based risk stratification and prediction of immune checkpoint inhibitor's efficacy for lung adenocarcinoma. Cancer Immunol. Immunother. 70 (6), 1705–1719. 10.1007/s00262-020-02817-z 33386920 PMC8139885

[B32] Yoshizawa A. Motoi N. Riely G. J. Sima C. S. Gerald W. L. Kris M. G. (2011). Impact of proposed IASLC/ATS/ERS classification of lung adenocarcinoma: Prognostic subgroups and implications for further revision of staging based on analysis of 514 stage I cases. Mod. Pathol. 24 (5), 653–664. 10.1038/modpathol.2010.232 21252858

[B33] Zhang L. Chen J. Cheng T. Yang H. Li H. Pan C. (2020). Identification of the key genes and characterizations of tumor immune microenvironment in lung adenocarcinoma (LUAD) and lung squamous cell carcinoma (LUSC). J. Cancer 11 (17), 4965–4979. 10.7150/jca.42531 32742444 PMC7378909

[B34] Zhao W. Mo H. Liu R. Chen T. Yang N. Liu Z. (2022). Matrix stiffness-induced upregulation of histone acetyltransferase KAT6A promotes hepatocellular carcinoma progression through regulating SOX2 expression. Br. J. Cancer 127, 202–210. 10.1038/s41416-022-01784-9 35332266 PMC9296676

[B35] Zhao Z. Xie L. Bourne P. E. (2019). Structural insights into characterizing binding sites in epidermal growth factor receptor kinase mutants. J. Chem. Inf. Model. 59, 453–462. 10.1021/acs.jcim.8b00458 30582689 PMC6441332

[B36] Zheng Q. Min S. Zhou Q. (2021). Identification of potential diagnostic and prognostic biomarkers for LUAD based on TCGA and GEO databases. Biosci. Rep. 41, BSR20204370. 10.1042/BSR20204370 34017995 PMC8182989

